# Supplementary material for: An integrated computational and experimental study uncovers FUT9 as a metabolic driver of colorectal cancer
Source: Mol Syst Biol. 2017 Dec 1;13(12):956. doi: 10.15252/msb.20177739 (PMC5740504; doi:10.15252/msb.20177739)
Supplement: Supplementary file 1 — Appendix [file MSB-13-956-s001.pdf]

Appendix

Table of Contents

Figure S1: FUT9 expression in adenoma vs. healthy
Figure S2: Glucose metabolism alterations following FUT9 knock-down
Figure S3: Glycosylation alterations following FUT9 knock-down
Figure S4: OCT4 expression in colon cancer following FUT9 knock-down
Figure S5: FUT9 copy number in M0 vs. M1 stages
Figure S6: MOMA scores for the knock-down of FUT9 in stages 1-4 using iMAT

Table EV1: Genes significantly downregulated in colon cancer
Table EV2: Genes with low copy number in colon tumors
Table EV3: Genes whose downregulation is negatively correlated with patient survival
Table EV4: MTA aggregated OTS scores for metabolic genes
Table EV5: MTA validation, genes KD collection in mouse/human
Table EV6: metabolic reactions afflicted by FUT9 loss in stage 3 colon cancer

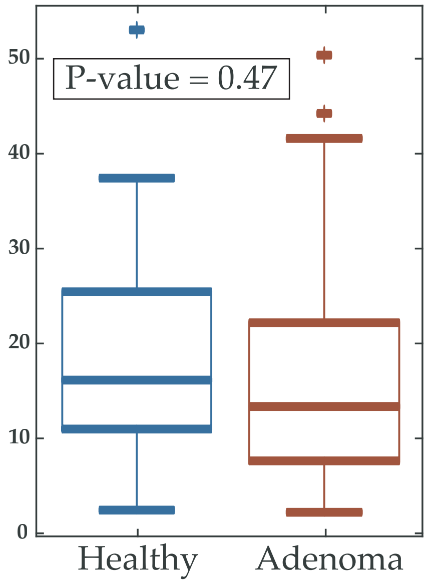

Appendix Figure S1- boxplots showing the expression of FUT9 in adenoma vs. healthy samples.

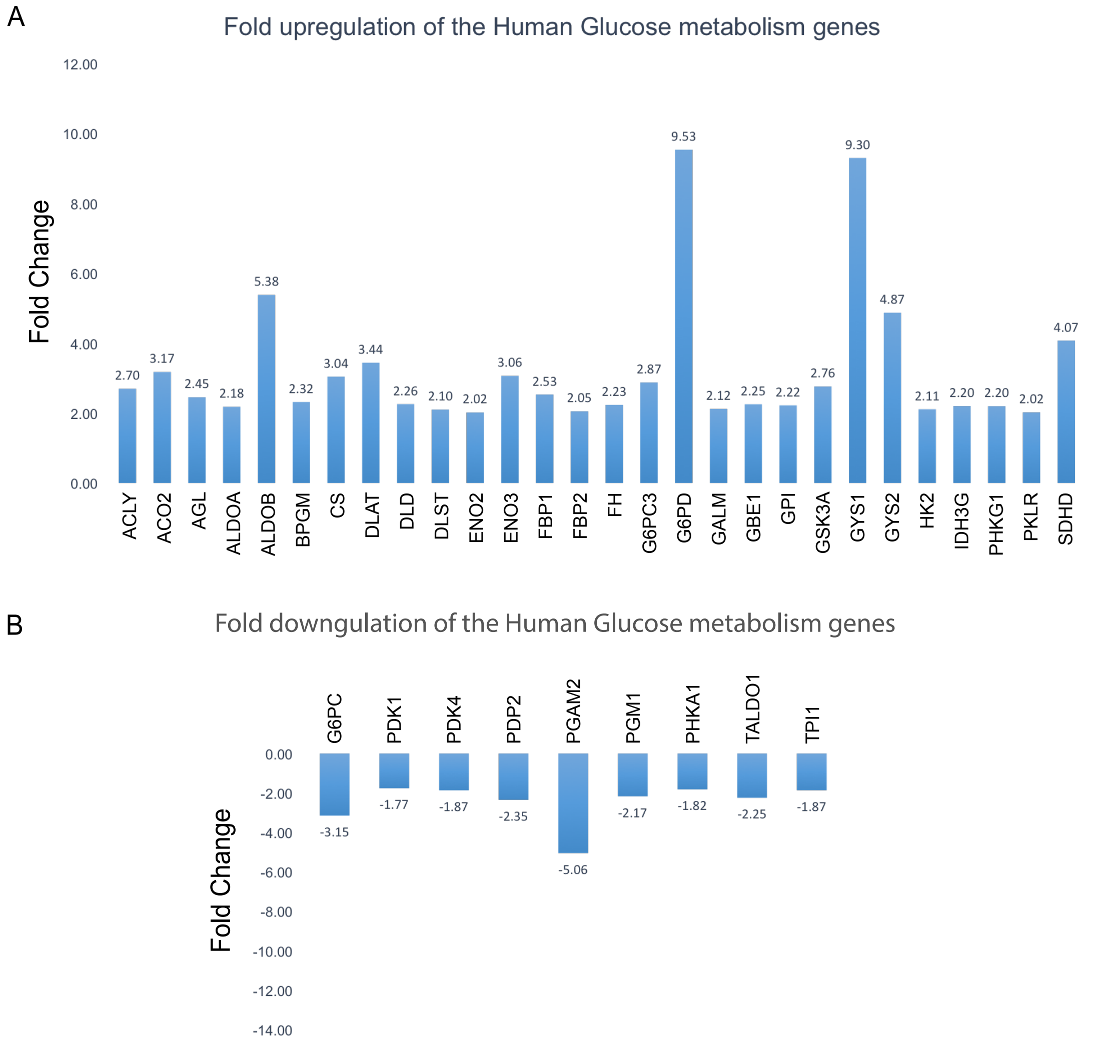

Appendix Figure S2- Bar graphs showing the fold change in gene expression after FUT9 shRNA knockdown in HCT116 cells as analyzed using the RT^2 Profiler human glucose metabolism PCR array. Fold change determined using the ΔΔCT method using shRFP as a non-targeting control. **(A)** Twenty eight genes showed a significant upregulation (≤ -2.0) when FUT9 is knocked down. **(B)** Nine genes showed significant downregulation (≥ 1.5) when FUT9 is knocked down.

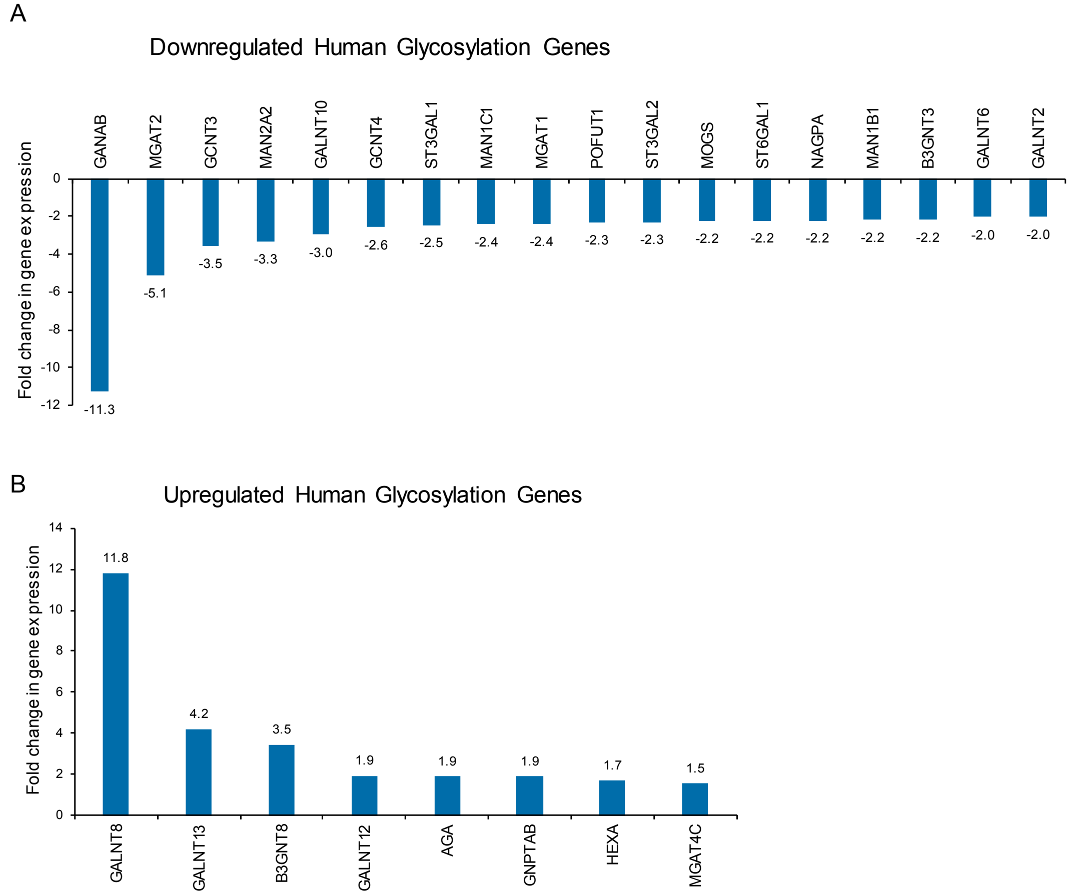

Appendix Figure S3: Bar graphs showing the fold change in gene expression after FUT9 shRNA knockdown in HCT116 cells as analyzed using the RT^2 Profiler human glycosylation PCR array. Fold change determined using the ΔΔCT method using shRFP as a non-targeting control. **(A)** Eighteen genes showed a significant downregulation (≤ -2.0) when FUT9 is knocked down. **(B)** Eight genes showed significant upregulation (≥ 1.5) when FUT9 is knocked down.

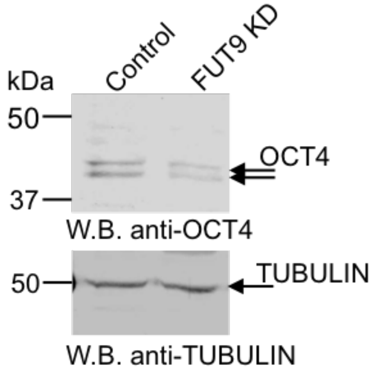

Appendix Figure S4- FUT9 knockdown suppresses OCT4 expression in colon cancer cells. Total cell lysates were prepared from human colon cancer cells, HCT116, with knocked down FUT9 expression (FUT9 KD) and from HCT116 cells transduced with control non-silencing shRNA (Control) using Lysis Buffer from the Proteome Profiler Stem Cell Array Kit (R&D). These cell lysates were resolved by SDS PAGE and OCT4 expression was analysed by Western blotting with anti-OCT4 antibody. Western blotting with anti-TUBULIN was used as a loading control. Images were captured using LI-COR Odyssey imager and Carestream software. Brightness and contrast were optimized using PowerPoint software.

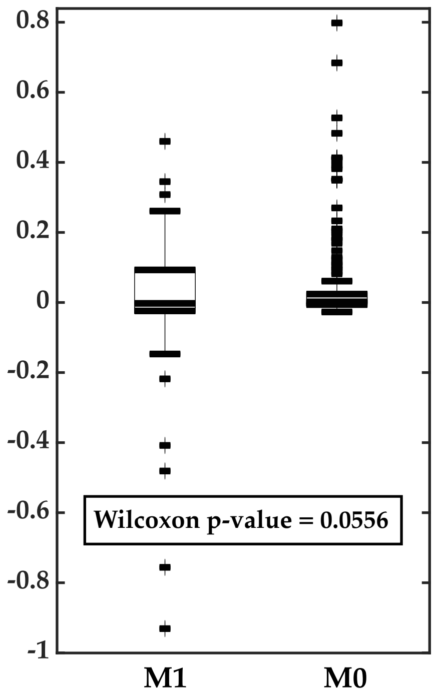

Appendix Figure S5- Boxplot showing FUT9 copy number for patients with M0 vs. M1 metastatic stage, using TCGA copy number data.

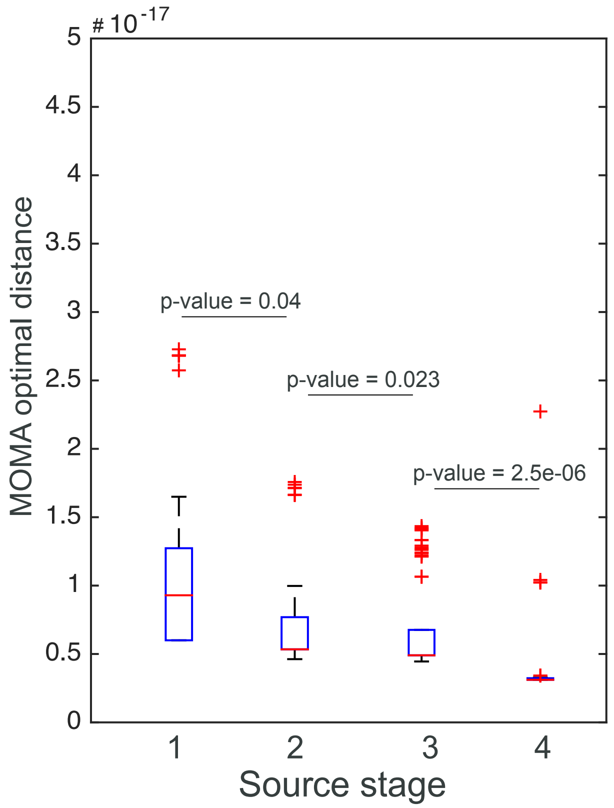

Appendix Figure S6- Boxplots sowing the MOMA scores obtained by the knock-down of FUT9 in stages 1-4 using iMAT algorithm to predict flux distributions.

Validating MTA through datasets of known perturbations
We set to evaluate the power of MTA to predict the true perturbation using human metabolic model and mammalian tissue/cell cultures. We hence applied MTA to 19 datasets with gene expression before and after performing known perturbation (Supplementary table S5), 3 of them are from human cell lines and 16 are from mice tissues. In all cases we used the recon1 human metabolic model. We find that

- For 4 out of 19 datasets MTA predicts the true perturbation in the top 5% (binomial p-value = 0.0112)
- For 8 out of 19 datasets MTA predicts the true perturbation in the top 10% (binomial p-value = 2.3718e-04),
- For 11 out of 19 datasets MTA predicts the true perturbation in the top 15% (binomial p-value = 1.7814e-05),
- For 13 out of 19 MTA predicts the true perturbation in the top 20% (binomial p-value = 5.8266e-06). In all datasets from human tissues MTA predicts the true perturbation in the top 20%.

These results show that MTA predictive power is robust for using mammalian tissue/cell cultures and human metabolic reconstruction. Knowing that MTA assigns high score to the true perturbation in most cases, it allows us to use it to identify driver genes by looking at genes that are repeatedly highly scores in many experiments.
