# Supplementary figures and images for: An integrated computational and experimental study uncovers FUT9 as a metabolic driver of colorectal cancer
Source: Mol Syst Biol. 2017 Dec 1;13(12):956. doi: 10.15252/msb.20177739 (PMC5740504; doi:10.15252/msb.20177739)

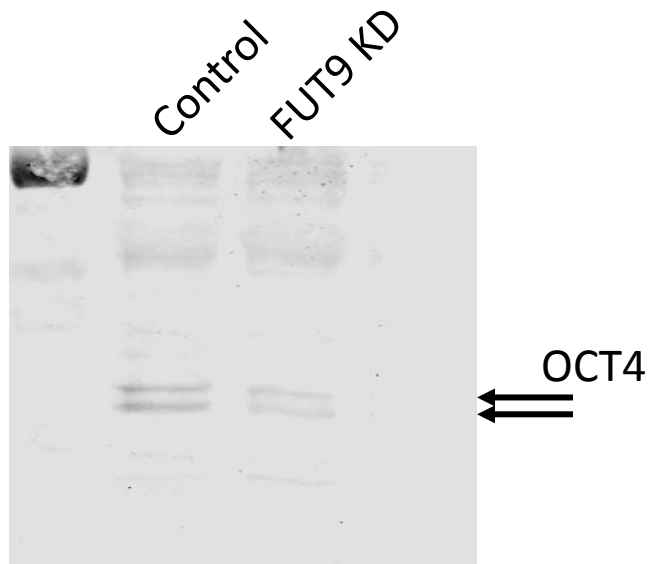

WB anti-OCT4

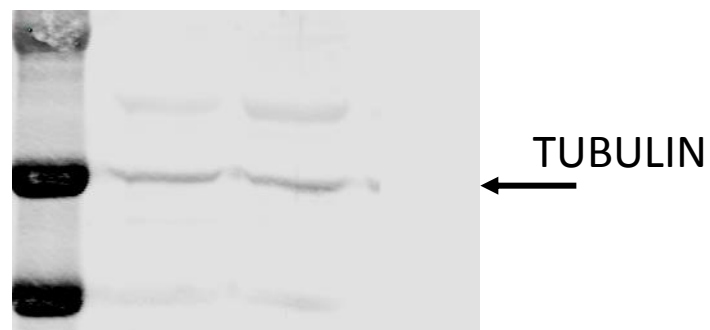

WB anti-TUBULIN

Supplement: Supplementary file 12 — Source Data for Appendix [file MSB-13-956-s012.pdf]
